# Aneurysm of Upper Limb Arteries in Children: Report of Five Cases

**DOI:** 10.1155/2020/9198723

**Published:** 2020-05-20

**Authors:** Ildar Nurmeev, Dmitry Osipov, Bruce Okoye

**Affiliations:** ^1^Department of Pediatric Surgery, Kazan Medical University, Kazan, Russia; ^2^Department of Cardiovascular Surgery, Children's Republican Hospital, Kazan, Russia; ^3^Department of Pediatric Surgery, St Georges Hospital NHS Trust, London, UK

## Abstract

**Background:**

Arterial aneurysm in children is rare.

**Aim:**

To present the description of case series of successful surgical treatment of upper limb aneurysms in children. The case series included 2 boys and 3 girls, with median age 3.3 years. One of them was a newborn with a true brachial artery aneurysm. Aneurysms were in the brachial (*n* = 3) and radial (*n* = 2) arteries. Two patients had idiopathic aneurysms. Two cases were associated with connective tissue dysplasia syndrome. One patient had a history of trauma. In 4 of 5 cases, there was a true aneurysm and in one a pseudoaneurysm. Diagnosis was carried out in all cases by using ultrasound, with arteriography in one case. All 5 children were operated on. Resection of the aneurysm and restoration of arterial patency was performed in 4 of 5 cases (ligation of the radial artery near the aneurysm in 1 case, aneurysm resection with end-to-end anastomosis in 1 case, resection with PTFE graft implantation in 1 case, and resection with implantation of an autovenous graft in 2 cases. *Complications*. Dysfunction and thrombosis of the PTFE graft required reoperation using an autovenous graft.

**Conclusion:**

Despite the rarity of the disease, timely and adequate surgical treatment of aneurysms of the arteries of the upper extremities in children is possible successfully in a specialized hospital.

## 1. Introduction

Arterial aneurysms are extremely rare in children. The true incidence is unknown. The development of pediatric aneurysms may be associated with concomitant processes, such as infections, injuries, connective tissue diseases, arteritis, or congenital vascular malformations [1, 2]. Only 5% of arterial aneurysms in children are found in the arteries of the upper limbs, and most of them are associated with systemic diseases [3].

Today, the cumulative number of cases described in patients younger than 12 years does not exceed two dozen. We present a case series of successful surgical treatment of upper limb aneurysms in children.

## 2. Case Presentation

A retrospective study of the surgical treatment of aneurysms of the arteries of the upper extremities in children treated in the Children's Republican Clinical Hospital, Kazan, was performed. The study was approved by the Local Ethic Committee of Kazan Medical University. Medical information and demographic data were obtained from medical records and hospital archives.

The case series included 2 boys and 3 girls, whose median age was 3.3 years. Aneurysms were in the brachial (*n* = 3) and radial (*n* = 2) arteries. Multiple aneurysms were not identified.

Two patients appeared to have idiopathic aneurysms. Two cases were associated with connective tissue dysplasia syndrome. One patient had a history of trauma. In 4 of 5 cases, there was a true aneurysm and in one a pseudoaneurysm.

A clinical and pathological classification of arterial aneurysms in children according to Sarkar is proposed. According to the classification, aneurysms in children are divided into 9 classes. Class I: associated with arterial infection, Class II: giant cell aortoarteritis, Class III: autoimmune connective tissue disease, Class IV: Kawasaki disease, Class V: Ehler-Danlos disease or Marfan syndrome, Class VI: other forms of noninflammatory middle membrane degeneration, Class VII: arterial dysplasia, VIII class: congenital idiopathic factors, and IX class: false aneurysm due to extravasal causes [4].

According to the classification of Sarkar, two of the cases reported were in class VIII, two in class VI, and one in class IX.

In two cases, paresthesia of the limb was observed due to nerve compression. In both cases, venous insufficiency of the limb with edema was present.

**Figure 1:**
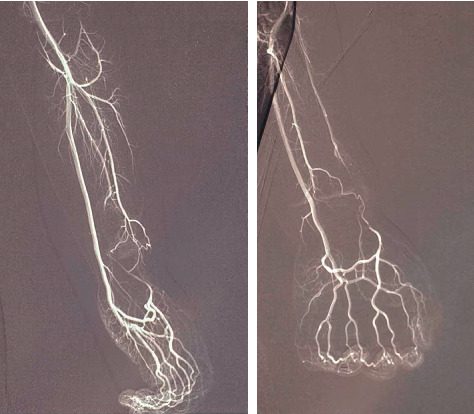
Radial artery aneurysm (arteriography image).

In all cases, diagnosis of aneurysms, preoperative examination, and postoperative results were reviewed using ultrasound. Arteriography was performed to confirm the diagnosis and determine the features of the aneurysm in only one case (Figure 1).

**Figure 2:**
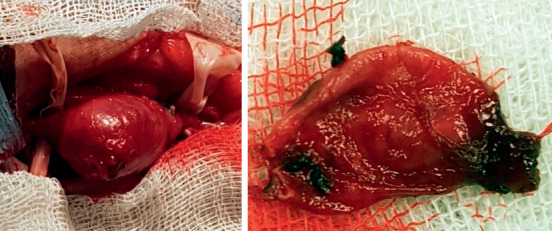
Brachial artery aneurysm.

All 5 children were operated on. Resection of the aneurysm and restoration of arterial patency was performed in 4 of 5 cases (see Figure 2).

**Figure 3:**
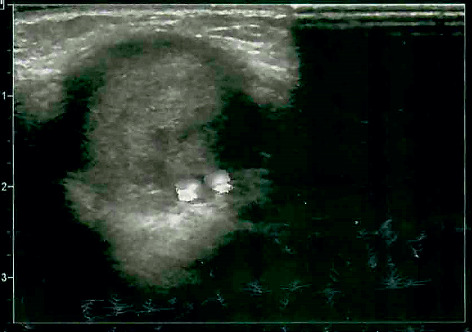
Radial artery aneurysm filled with thrombotic masses (an ultrasound image).

In one case, ligation of the radial artery near the aneurysm was made. Before the operation, the Allen test was performed, which demonstrated good development of collateral blood flow [5]. The ligation procedure did not affect the blood supply to the hand after surgery. In the same case, the aneurysmal cavity was found to be filled with thrombotic masses (see Figure 3).

**Figure 4:**
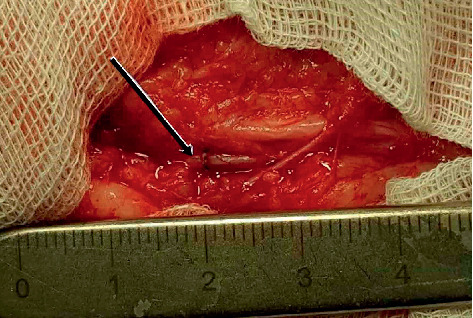
Brachial artery aneurysm resected and end-to-end anastomosis performed.

In one case, a surgical restoration of brachial artery patency was performed with an end-to-end anastomosis. This was feasible since the distance between the ends of the artery was adequate for the connection (see Figure 4).

**Figure 5:**
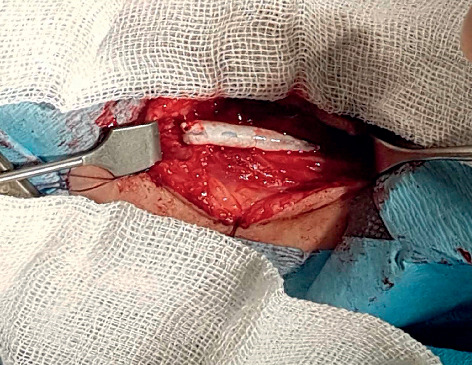
Aneurysm of the brachial artery resected with PTFE graft implantation.

In one case, a brachial artery restoration operation was performed using a polytetrafluoroethylene prosthesis (PTFE graft) (see Figure 5). In this case, a complication was noted on postoperative day 5. Dysfunction and thrombosis of the prosthesis required reoperation and implantation of an autovenous graft.

In two cases, surgical treatment was performed by primary implantation of an autovenous graft. In one of these cases, a segment of a vein from the forearm of the operated limb was used for implantation. In the second case, the graft was obtained from the great saphenous vein from the lower limb.

## 3. Discussion

Aneurysm of the arteries of the upper limbs in children is a rare pathology. Usually the descriptions refer to individual cases or small series of cases.

Davis et al. reported 7 cases of upper extremity arterial aneurysms with the youngest child aged 2 months [2]. Sarkar et al. reported 4 aneurysms of the upper limb (one of them in the brachial artery of a child of two weeks of age) [4]. Reviews by other authors report a reference in the world literature of a total of up to 14 different clinical cases in children under the age of 12 years [1, 3, 6].

Cases of successful diagnosis and treatment of brachial artery aneurysms in newborns are vanishingly rare.

The choice of surgical procedure depends on the type of aneurysm and the length of the arterial defect. In the case of pseudoaneurysms, damage to the artery wall is minimal and it is even possible to suture the defect [7, 8].

It is possible to perform end-to-end anastomosis in most pseudoaneurysms [9, 10]. In cases of true aneurysms, venous graft implantations are performed more often [2]. The case of a newborn child with a true brachial artery aneurysm treated surgically in this report is unique.

The unique feature of the series of cases described in the article was that all children were operated on. There were no cases treated by observation alone. The preferred type of operation is an end-to-end artery repair. When this is not possible, a good result is provided by the implantation of a venous graft. In one case, however, a complication of the operation was noted—ectasia of the implanted venous graft. Opinions regarding the timing of surgical treatment vary. Some authors believe that observation tactics are possible with subsequent surgical correction at an older age for all cases with an uncomplicated course [1]. Other experts believe that the operation is preferable immediately after the diagnosis in order to avoid limb ischemia [11].

The cases described were characterized by the progression of the disease with an increase in the volume of the aneurysm. In addition, in two of the cases, there was an impact on the nerves of the upper limb with neurological symptoms. Patients also had signs of venous insufficiency of the limb such as edema and ischemia, as well as impaired function of the limb.

**Figure 6:**
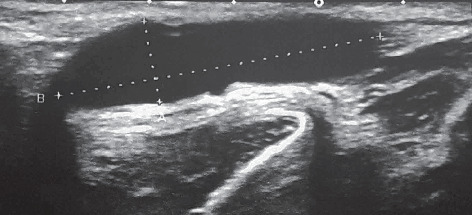
Autovenous graft dilated (an ultrasound image).

All operated patients are under the observation of specialists. In two cases (class IX aneurysms), significant dilation of venous grafts have been diagnosed (see Figure 6). Reoperation has not been required.

Follow-up of patients for 1–3 years showed good results.

## 4. Conclusion

Despite the rarity of the disease, timely and adequate surgical treatment of aneurysms of the arteries of the upper extremities in children is possible successfully in a specialized hospital.
